# Mitochondrial K_ATP_ channel involvement in angiotensin II-induced autophagy in vascular smooth muscle cells

**DOI:** 10.1007/s00395-014-0416-y

**Published:** 2014-05-22

**Authors:** Kang-Ying Yu, Ya-Ping Wang, Lin-Hui Wang, Yang Jian, Xiao-Dong Zhao, Jing-Wei Chen, Koji Murao, Wei Zhu, Liang Dong, Guo-Qing Wang, Guo-Xing Zhang

**Affiliations:** 1 Department of Physiology, Medical College of Soochow University, 199 Ren-Ai Road, Dushu Lake Campus, Suzhou Industrial Park, Suzhou, 215123 People’s Republic of China; 2 Department of Clinical Laboratory, Wuxi Second People’s Hospital, 68 Zhong-Shang Road, Wuxi, 214002 People’s Republic of China; 3 Department of Internal Medicine, The Affiliated Suzhou Chinese Traditional Medicine Hospital, Nanjing University of Chinese Medicine, 18 Yang-Su Road, Suzhou, 215003 People’s Republic of China; 4 Department of Clinical Laboratory, Faculty of Medicine, Kagawa University, 1750-1 Ikenobe Miki-cho, Kita-gun, Kagawa, 761-0793 Japan; 5 Department of Internal Medicine, The Second Affiliated Hospital, High-tech zone Hospital, Soochow University, 28 Kang-Fu Alley, Suzhou High-tech Zone Hu-Shu-Guan Town, Suzhou, 215151 People’s Republic of China; 6 Department of Pathophysiology, Medical College of Soochow University, 199 Ren-Ai Road, Dushu Lake Campus, Suzhou Industrial Park, Suzhou, 215123 People’s Republic of China

**Keywords:** Angiotensin II (Ang II), NADPH oxidase, Mitochondrial ATP-sensitive potassium channels, Autophagy, LC3-II

## Abstract

Autophagy has emerged as a powerful process in the response to cellular injury. The present study was designed to investigate signal transduction pathways in angiotensin II (Ang II)-induced autophagy. Rat vascular smooth muscle cells (VSMCs) were stimulated with different doses of Ang II (10^−9^–10^−5^ mol/L) for different time periods (6–72 h). Incubation with Ang II increased the production of reactive oxygen species (ROS), increased the LC3-II to LC3-I ratio, increased beclin-1 expression, and decreased SQSTM1/p62 expression in a dose- and time-dependent manner. In addition, Ang II increased autophagosome formation. Increased ROS production induced by Ang II was inhibited by Ang II type 1 receptor (AT1) blockers (Olmesartan and Candesartan, ARB), a NADPH Oxidase inhibitor (apocynin), and mitochondrial K_ATP_ channels inhibitor (5-hydroxydecanoate, 5HD). Ang II (10^−7^ mol/L, 48 h)-induced increase in the LC3-II to LC3-I ratio, the formation of autophagosomes, expression of beclin-1 and decrease in the expression of SQSTM1/p62 were also inhibited by pretreatment with 3-methyladenine or bafilomycin A1 (inhibitors of autophagy), olmesartan and candesartan (in dose-dependent manners), apocynin, 5HD, and siRNA Atg5. Our results indicate that Ang II increases autophagy levels via activation of AT1 receptor and NADPH oxidase. Mitochondrial K_ATP_ channels also play an important role in Ang II-induced autophagy. Our results may provide a new strategy for treatment of cardiovascular diseases with Ang II.

## Introduction

Angiotensin II (Ang II) is a bioactive peptide of the renin–angiotensin–aldosterone system and plays an important role in cell growth, differentiation, apoptosis, induction of pro-inflammatory cytokines, and fibrogenesis [10, 25, 40]. An increasing number of reports have demonstrated that an increase of Ang II in the vascular system is implicated in the pathogenesis of cardiovascular diseases, such as hypertension, atherosclerosis, and diabetes [12, 17]. It has been reported that Ang II activates membrane-bound NADPH oxidase to produce reactive oxygen species (ROS) leading to an increase in mitochondrial-derived ROS release, thereafter activating downstream signaling pathways [18, 27, 56].Currently, ROS is generally recognized as one of the key second messengers that mediate Ang II-induced cell injury [37, 42]. Therefore, antioxidant application is one novel strategy in the treatment of clinical patients with cardiovascular diseases induced by high Ang II levels.

Traditionally, autophagy is considered to play an important role in maintaining cellular homeostasis, differentiation, and tissue remodeling through the formation of the autophagosome that digests macromolecules into amino acids. Paradoxically, the role of autophagy in cell survival is still controversial. Nutrient depletion induces autophagy which provides amino acids for synthesis of essential proteins, thus prolonging cell survival [22]. In addition, it has also been reported that autophagy can counteract cell apoptosis [38]. However, several other studies have shown that autophagy serves as an alternative form of cell death [6, 9], and ROS is reported to play an essential role in the induction of autophagy [43, 52]. Recently, it has been postulated that the role of autophagy is dependent upon the induction levels of autophagy. A mild induction of autophagy by ROS may exert cytoprotective effects, whereas a massive induction of autophagy by ROS may cause excessive self-digestion of cell components and lead to cell death [16]. Yadav et al. [54] recently reported that Ang II induces autophagy in podocytes, which may contribute to Ang II-induced renal injury. Whether Ang II also induces autophagy in vascular smooth muscle cells (VSMCs) and the underlying mechanism for this phenomenon is still unknown.

Mitochondrial ATP-sensitive potassium (K_ATP_) channels, located in the inner mitochondrial membrane, have been extensively investigated [2, 3, 46]. The protective role of mitochondrial K_ATP_ channels has mainly focused on preconditioning or post-preconditioning [5, 14, 15, 35]. Our previous report has demonstrated that Ang II-induced preconditioning is also mediated by mitochondrial K_ATP_ channels [19]. In addition, our study has shown that Ang II induces mitochondrial K_ATP_ channels to open, depolarizes mitochondrial membrane potential, increases ROS generation by the mitochondria, and mediates the downstream signal transduction in VSMC [18]. However, the role of mitochondrial K_ATP_ channels in the induction of autophagy has not been characterized.

Based on these reports, our hypothesis is that Ang II activates NADPH oxidase via the AT1 receptor, opens mitochondrial KATP channels, increases ROS generation, and results in the induction of autophagy in VSMC, and that this process may play a role in Ang II-induced atherosclerosis.

## Methods

### Cell culture

Rat vascular smooth muscle cells (VSMCs) were prepared by explantation of the descending thoracic aorta of 4-week-old male rats under anesthesia (10 % chloral hydrate; 350 mg/kg ip.) and were maintained in Dulbecco’s modified Eagle’s medium supplemented with 10 % (v/v) fetal bovine serum as previously reported [18]. All of the cells were incubated at 37 °C in 5 % CO_2_ in air. VSMCs were authenticated using immunohistochemical staining for α-smooth muscle actin (Millipore, CBL171). Cultures showing more than 95 % staining for α-actin between passage three and seven were used. All experimental procedures were performed according to the guidelines for the care and use of animals established by Soochow University and conformed to the Guide for the Care and Use of Laboratory Animals published by the US National Institutes of Health (NIH Publication No. 85-23, revised 1996).

### Measurement of ratio of LC3-II to LC3-I, beclin-1 and SQSTM1/p62 expressions in VSMCs

Expression of beclin-1 and SQSTM1/p62 and the ratio of LC3-II to LC3-I were analyzed by western blotting as previously described [18]. Briefly, VSMCs were starved for 12 h, and incubated with Ang II for different periods of time (6–72 h, control group was starved for 72 h) or at varying concentration (10^−9^–10^−5^ mol/L) or treated with diazoxide [18] (K_ATP_ channels opener, for 24 and 48 h, 2 × 10^−4^ mol/L) with or without pretreatment with 3-methyladenine (3-MA, an inhibitor of autophagy, 2 × 10^−3^ mol/L), bafilomycin A1(Baf, an inhibitor of autophagy, 1 × 10^−4^ mol/L), olmesartan and candesartan (10^−6^–10^−4^ mol/L, ARB, AT1 receptor blocker), apocynin (NADPH Oxidase inhibitor, 1 × 10^−4^ mol/L), 5-hydroxydecanoate (5-HD, mitochondrial K_ATP_ channels inhibitor, 1 × 10^−4^ mol/L), and rapamycin (autophagy inducer, 10 mg/L), and the cells were directly lysed in the cell lysis buffer consisting of 20 mM Tris–HCl, pH 7.4, 140 mM NaCl, 1 % Triton-X, 10 % Glycerol, 1 mM β-glycerophosphate, 1 mM sodium orthovanadate and protease inhibitor cocktail tablets (Roche Diagnostics). The lysate was subjected to sonication (20 s, three times) on ice, followed by centrifugation for 30 min at 1,000×*g* to remove cellular debris. The protein concentration of the supernatant was assessed with a protein assay kit (Bio-Rad). Equal amounts of protein from each sample were resolved by 10 % SDS-PAGE and the proteins were transferred onto PVDF membranes (Hybond TM-ECL; Amersham Pharmacia Biotech). The membranes were blocked for 2 h at room temperature with 5 % skimmed milk in PBS and 0.1 % Tween 20. The blots were incubated overnight with a 1:1,000 dilution of the following primary antibodies: anti-LC3-II (Abcam Corporation, abc62721), beclin-1 (Epitomics, Inc. 2026-1), SQSTM1/p62 (Abcam Corporation, abc109012), and anti-GAPDH (Santa Cruz Biotech, AP0063) followed by incubation for 1 h with a secondary antibody (HRP-conjugated anti-rabbit IgG; 1:2,000, Abgent, LP1001a). Immunoreactive bands were visualized using enhanced chemiluminescence (ECL; Amersham Pharmacia Biotech) and quantified by NIH image software. Data were normalized to GAPDH. All of the data were presented as fold change of the control group.

### Immunofluorescence detection of autophagosomes in VSMCs

Immunofluorescent detection of LC3 association with autophagosomes was carried out as previously described [50]. In brief, the VSMCs were fixed with 1:1 methanol and acetone, washed with PBS and then incubated in PBS containing 0.1 % Triton-X-100 for 10 min. After washing with PBS again, the cells were incubated at room temperature for 1 h in a blocking solution composed of PBS containing 2 % non-fat milk. Cells were then incubated overnight at 4 °C in blocking solution containing mouse anti-LC3-II (Abcam Corporation, abc62721; diluted to 1:200), anti-α-smooth muscle actin antibody (Millipore Corporation, CBL171; diluted to 1:1,000). Next, cells were incubated in a blocking solution containing Cy3-conjugated donkey anti-mouse immunoglobulin G antibody (Jackson Immuno Research Laboratories, 715-165-150) at a dilution of 1:600 and fluorescein-isothiocyanate conjugated donkey anti-rabbit immunoglobulin G antibody (Jackson Immuno Research Laboratories, 711-095-152) at a dilution of 1:800 for 2 h at room temperature. After washing with PBS, nuclei were stained with 40, 60-diamidino-2-phenylindole (DAPI; Sigma, 28718-90-3) for 10 min at room temperature. Finally, immunostained cells were rinsed with PBS and examined under a fluorescence microscope (Eclipse TE 2000-U; Nikon, Tokyo, Japan) equipped with a filter system or a confocal microscope (C1 plus sci; Nikon). Microslips were randomly scanned with the confocal microscope, and six to eight microslips were analyzed for each group. Twenty cells on each microslip were randomly analyzed for the presence and number of autophagosomes in a double-blind fashion, and data were presented as an average of each group.

### Quantitative analysis of the VSMCs viability

Analysis of VSMC viability was performed after Ang II (10^−7^ mol/L) stimulation for 24–72 h as previously reported [50]. Briefly, MTT [3-(4,5-dimethylthiazol-2-yl)-2,5-diphenyl tetrazolium bromide, Sigma, M-0283] solution was added to the culture medium (final concentration was 5 mg/ml) 4 h before the end of treatment. The reaction was stopped by the addition of 10 % acidified SDS (100 ul) to the cell culture. The absorbance value was measured at 570 nm using an automatic multi-well spectrophotometer (Bio-Rad, Richmond, CA, USA). Data were represented as fold change compared with the control group which had remained in the culture for 24 h, but was not treated.

### Measurement of ROS production

ROS levels in VSMCs were determined by the dihydroethidium (DHE) staining method as previously described [18]. After treatment with Ang II (10^−7^ mol/L), with or without 3-MA (2 × 10^−3^ mol/L), olmesartan (10^−4^ mol/L), candesartan (10^−4^ mol/L), apocynin (10^−4^ mol/L), or 5-HD (10^−4^ mol/L), cells were incubated with 5 uM DHE for 10 min at 37 °C. VSMCs were washed with PBS and DHE fluorescence images were visualized with a confocal microscope (C1 plus sci; Nikon) by excitation at 488 nm and emission at 595 nm for the detection of the oxidized DHE product ethidium (shown in red). Densitometry analysis was performed after various treatments. Data were represented as fold change compared with the control group as described previously [55].

### Knock-down of Atg5

Small interfering RNAs (siRNA) targeting the following mRNA were utilized: Atg5 (1) sense 5′-GACGCUGGUAACUGACAAATT-3′ and antisense 5′-UUUGUCAGUUACCAGCGUCTT-3′, (2) sense 5′-GGCCUUUCAUUCAGAAGCUTT-3′ and antisense 5′-AGCUUCUGAAUGAAAGGCCTT-3′. Negative control siRNA was as follows: sense 5′-UUCUCCGAACGUGUCACGUTT-3′, antisense 5′-ACGUGACACGUUCGGAGAATT-3′ were designed and synthesized by GenePharma (Shanghai, China). For transfection, cells were plated in 9-cm dishes at 30 % confluence, and siRNA duplexes (200 nM) were introduced into the cells using Lipofectamine 2000 (Invitrogen, 11668-019, Shanghai, China) according to the manufacturer’s recommendations. Protein expression of Atg5 was verified by western blot analysis (anti-Atg5 antibody, Epitomics, Inc. 3447-1). After transfection, VSMCs were stimulated with Ang II (10^−7^ mol/L, 24 h) similar to previous experiments in the present study, and the ratio of LC3-II to LC3-I, expression of SQSMT1/p62 and formation of autophagosomes were determined.

### Statistical analysis

All data were presented as the mean ± SEM. Statistical significance between more than two groups was tested using two-way ANOVA followed by the Newman-Keel test or an unpaired two-tail Student’s *t* test. *P* values of <0.05 were considered statistically significant.

## Results

### Ang II time- and dose-dependently increased the LC3 II to LC3-I ratio, beclin-1 expression, and decreased SQSTM1/p62 protein expression

**Fig. 1:**
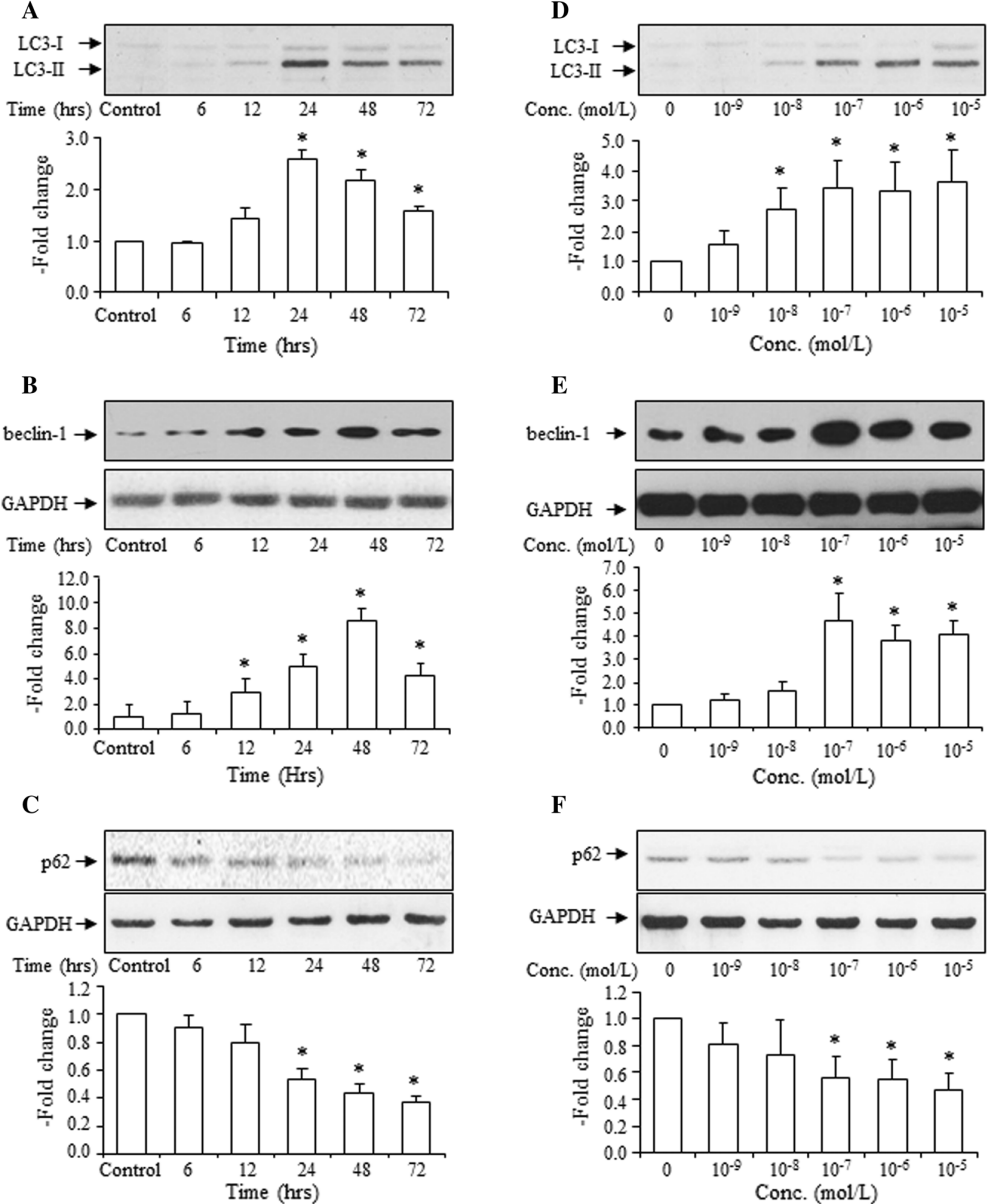
Time course and dose response of Ang II effects on the LC3-II to LC3-I ratio, expression of beclin-1 and SQSMT1/p62 by VSMCs. Time course (**a**) and dose response (**d**) of Ang II effects on the LC3-II to LC3-I ratio, *top* representative blots of LC3-II and LC3-I after treatment with Ang II, *bottom* densitometry analysis of the ratio of LC3-II to LC3-I. Time course (**b**) and dose response (**e**) of Ang II on beclin-1 expression, *top* representative blots of beclin-1 and GAPDH after treatment with Ang II, *bottom* densitometry analysis of beclin-1. Time course (**c**), and dose response (**f**) of Ang II on SQSMT1/p62 expressions, *top* representative blots of SQSMT1/p62 and GAPDH after treatment with Ang II, *bottom* densitometry analysis of SQSMT1/p62 and GAPDH. Ang II (10^−7^ mol/L) stimulates for 6, 12, 24, 48 and 72 h. Ang II (10^−9^–10^−5^ mol/L) stimulates for 24 h. Control group is expressed as 1. Data are presented as mean ± SEM (*n* = 4–6). *p < 0.05 vs. control

VSMCs were incubated with Ang II (10^−7^ mol/L) for 6, 12, 24, 48 and 72 h. From 24 to 72 h, the LC3-II to LC3-I ratio (Fig. 1a) and expression of beclin-1 (Fig. 1b) were significantly increased, and expression of SQSTM1/p62 (Fig. 1c) was significantly decreased.

VSMCs were incubated with Ang II (10^−9^–10^−5^ mol/L) for 48 h. Statistical analysis showed that Ang II stimulation increased the LC3-II to LC3-I ratio (Fig. 1d) and expression of beclin-1 (Fig. 1e), and decreased expression of SQSTM1/p62 (Fig. 1f) in a dose-dependent manner, with the optimal dose at 10^−7^ mol/L.

### Effect of Ang II on VSMCs viability

**Fig. 2:**
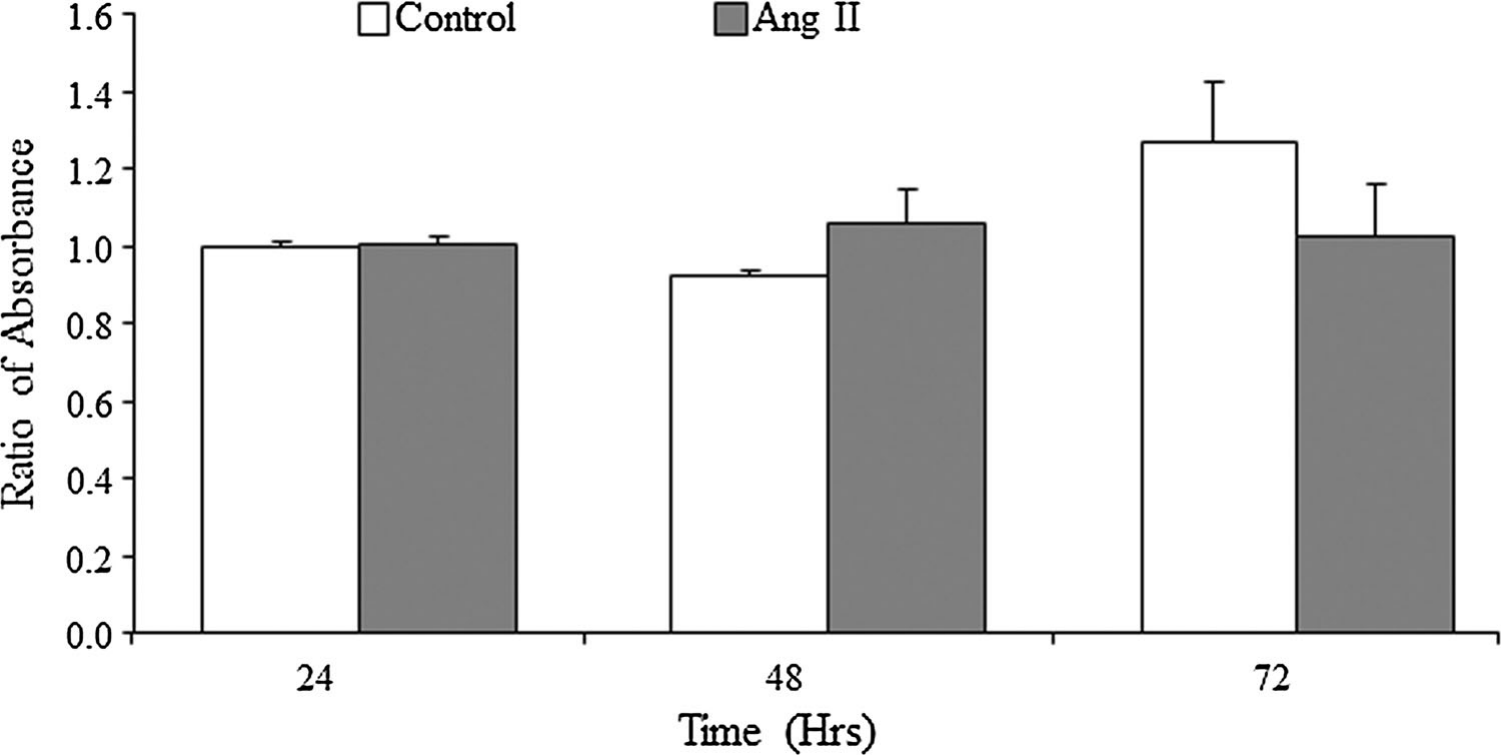
Effects of Ang II on VSMCs viability. VSMCs are treated with Ang II (10^−7^ mol/L) for 24–72 h, and viability was analyzed by MTT assay. Data from each group are expressed as the ratio of optical density to the 24-h control group and are presented as mean ± SEM (*n* = 8)

To confirm the effects of Ang II on cell survival, VSMCs viability was examined using the MTT assay. The results showed that Ang II stimulation for 24–72 h (10^−7^ mol/L) did not affect cell viability (Fig. 2).

### Effects of 3-methyladenine (3-MA) or bafilomycin A1 (Baf) on Ang II-induced increase of LC3-II to LC3-I ratio, beclin-1 expression, and decrease of SQSMT1/p62 expression

**Fig. 3:**
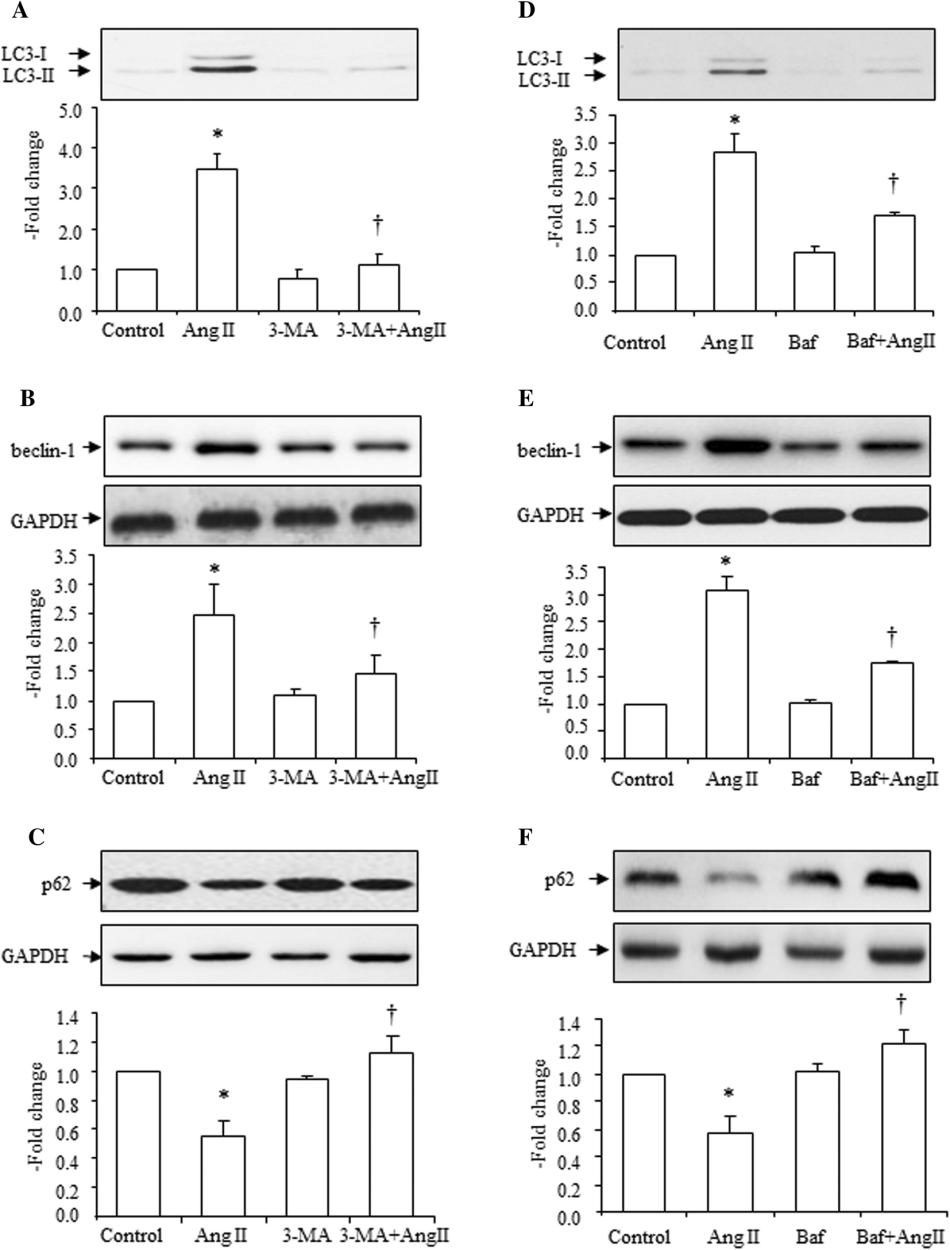
Effects of 3-methyladenine [3-MA, (2 × 10^−3^ mol/L)] or bafilomycin A1 [Baf, (10^−4^ mol/L)] on Ang II (10^−7^ mol/L)-induced increase in LC3-II, beclin-1 and decrease in SQSMT1/p62 expressions. Effects of 3-MA (**a**) and Baf (**d**) on Ang II-induced increase in the LC3-II to LC3-I ratio, *top* representative blots of LC3-II and LC3-I expression after treatment with Ang II, *bottom* densitometry analysis of the ratio of LC3-II to LC3-I. Effects of 3-MA (**b**) and Baf (**e**) on Ang II-induced increase in beclin-1 expression, *top* representative blots of beclin-1 and GAPDH after treatment with Ang II, *bottom* densitometry analysis of beclin-1 and GAPDH. Effects of 3-MA (**c**) and Baf (**f**) on Ang II-induced decrease in SQSMT1/p62 expression, *top* representative blots of SQSMT1/p62 and GAPDH after treatment with Ang II, bottom: densitometry analysis of SQSMT1/p62 and GAPDH. Control group is expressed as 1. Data are presented as mean ± SEM (*n* = 6–8). *p < 0.05 vs. control. ^†^ *p* < 0.05 vs. Ang II

Thirty minutes before Ang II (10^−7^ mol/L) stimulation, an autophagy inhibitor, 3-MA or Baf was added to the cells. The results showed that 3-MA completely suppressed the Ang II-induced increase of the LC3-II to LC3-I ratio (Fig. 3a). An increase in beclin-1 expression caused by Ang II was also inhibited by 3-MA (Fig. 3b). A decrease in SQSMT1/p62 expression by Ang II was also reversed by 3-MA (Fig. 3c). 3-MA alone did not significantly affect the LC3-II to LC3-I ratio or expression of beclin-1 and SQSMT1/p62 (Fig. 3a, b, c).

Similar results were also obtained in the Baf pretreatment experiments. Baf markedly suppressed Ang II-induced increase of the LC3-II to LC3-I ratio (Fig. 3d). An increase in beclin-1 expression caused by Ang II was also inhibited by Baf (Fig. 3e). A decrease in SQSMT1/p62 expression caused by Ang II was also reversed by Baf (Fig. 3f). Baf alone did not significantly affect the LC3-II to LC3-I ratio or expression of beclin-1 and SQSMT1/p62 (Fig. 3d–f).

### Effects of olmesartan, candesartan, apocynin and 5-HD on Ang II-induced increase of ROS production

**Fig. 4:**
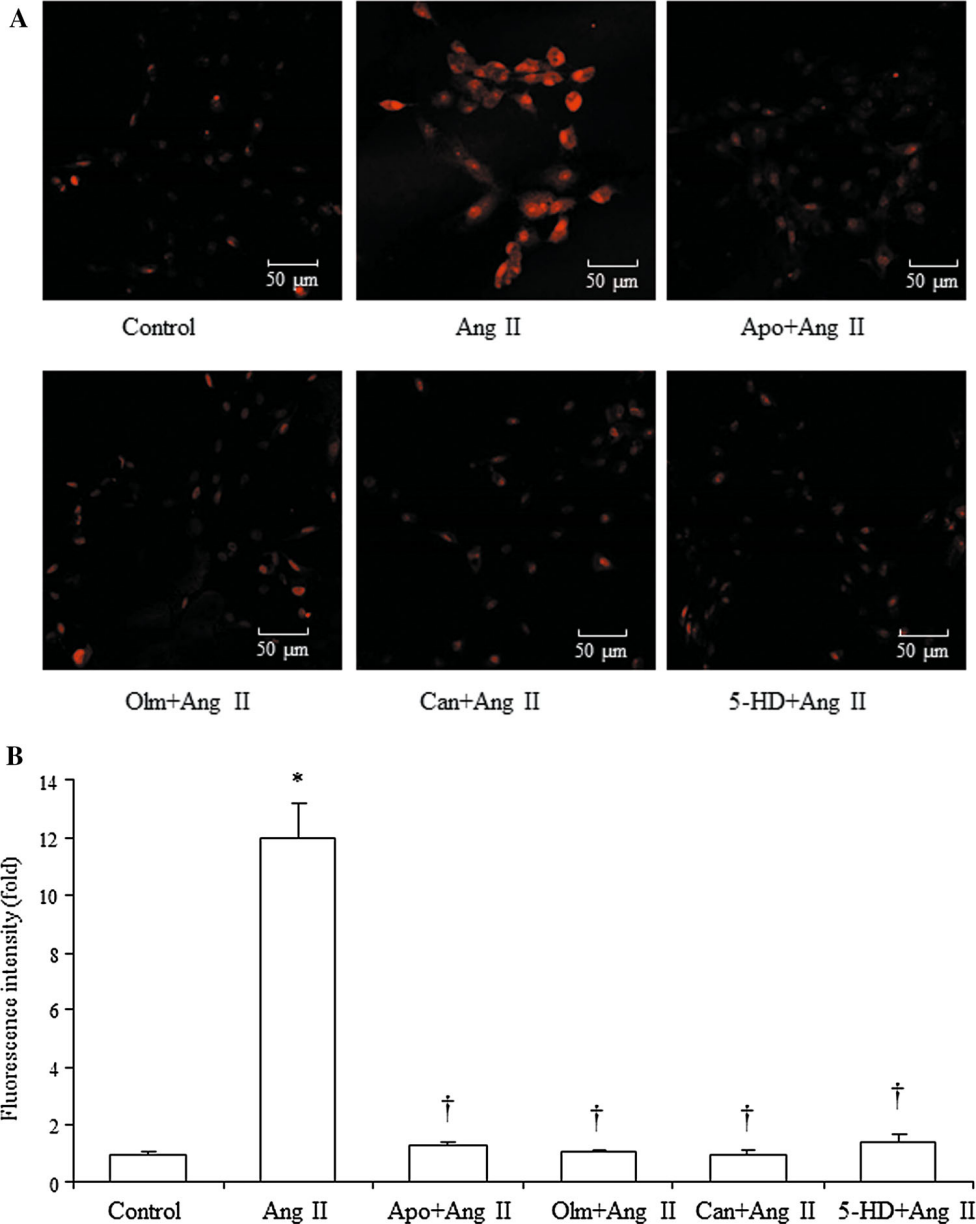
Effects of olmesartan (Olm, 10^−4^ mol/L), Candesartan (Can, 10^−4^ mol/L), apocynin (10^−4^ mol/L), and 5-HD (10^−4^ mol/L) on Ang II (10^−7^ mol/L)-induced increase in ROS production. **a** Representative fluorescence images of VSMCs after stimulated with Ang II for 48 h with or without treatment with Olm, Can, Apo and 5-HD. **b** ROS levels shown by intensity of ethidium (oxidized form of DHE) fluorescence in VSMCs in each group. Data are presented as mean ± SEM (*n* = 6). *p < 0.05 vs. control. ^†^ *p* < 0.05 vs. Ang II

When VSMCs were incubated with Ang II (10^−7^ mol/L) for 48 h, ROS production was significantly increased (Fig. 4a, b). Olmesartan (10^−4^ mol/L), candesartan (10^−4^ mol/L), apocynin (10^−4^ mol/L), and 5-HD (10^−4^ mol/L) all markedly reduced Ang II-induced increase of ROS generation (Fig. 4a, b).

### Effects of olmesartan, candesartan and apocynin on Ang II-induced increase of the LC3-II to LC3-I ratio, beclin-1 expression, and decrease of SQSMT1/p62 expression

**Fig. 5:**
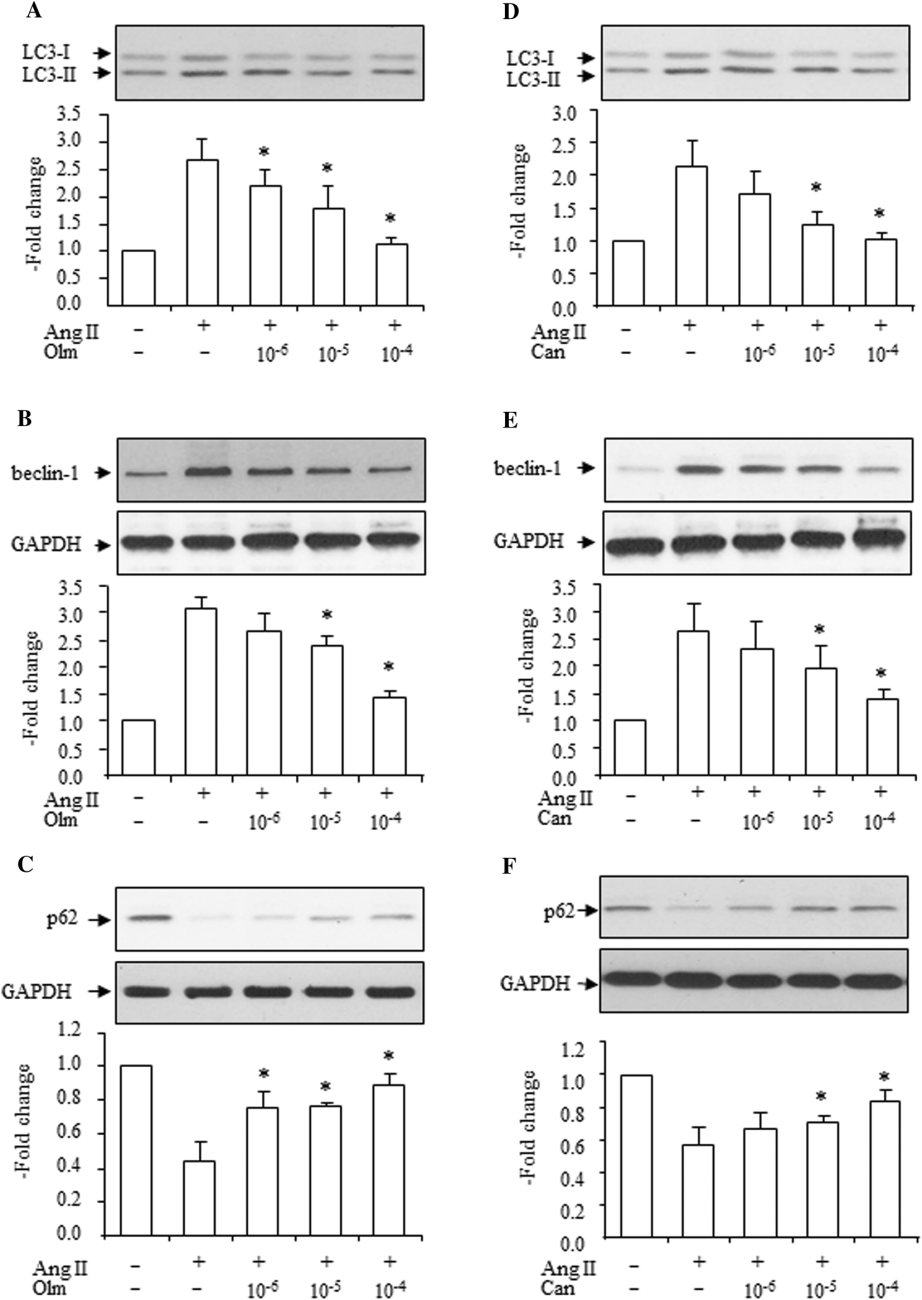
Effects of olmesartan (Olm, 10^−6^–10^−4^ mol/L) and candesartan (Can, 10^−6^–10^−4^ mol/L) on Ang II (10^−7^ mol/L)-induced increase in LC3-II, beclin-1 and decrease in SQSMT1/p62 expressions. Effects of Olm (**a**) and Can (**d**) on Ang II-induced increase in LC3-II to LC3-I ratio, *top* representative blots of LC3-II and LC3-I expression after treatment with Ang II, *bottom* densitometry analysis of the LC3-II to LC3-I expression ratio. Effects of Olm (**b**) and Can (**e**) on Ang II-induced increase in beclin-1 expression, *top* representative blots of beclin-1 and GAPDH after treatment with Ang II, *bottom* densitometry analysis of beclin-1 and GAPDH. Effects of Olm (**c**) and Can (**f**) on Ang II-induced decrease in SQSMT1/p62 expression, *top* representative blots of SQSMT1/p62 and GAPDH expression after treatment with Ang II, *bottom* densitometry analysis of SQSMT1/p62 and GAPDH expression. Control group is expressed as 1. Data are presented as mean ± SEM (*n* = 5–6). *p < 0.05 vs. Ang II

The AT1 receptor blocker, olmesartan (10^−6^–10^−4^ mol/L), dose-dependently blocked Ang II (10^−7^ mol/L)-induced increases of the LC3-II to LC3-I ratio (Fig. 5a), increase in beclin-1 expression (Fig. 5b), and decrease in SQSMT1/p62 expression (Fig. 5c).

Another AT1 receptor blocker, candesartan (10^−6^–10^−4^ mol/L), also dose-dependently blocked Ang II (10^−7^ mol/L)-induced increases in the LC3-II to LC3-I ratio (Fig. 5d), increase in beclin-1 expression (Fig. 5e), and decreases in SQSMT1/p62 expression (Fig. 5f). These data suggest that Ang II-induced autophagy occurs via AT1 receptor activation.

**Fig. 6:**
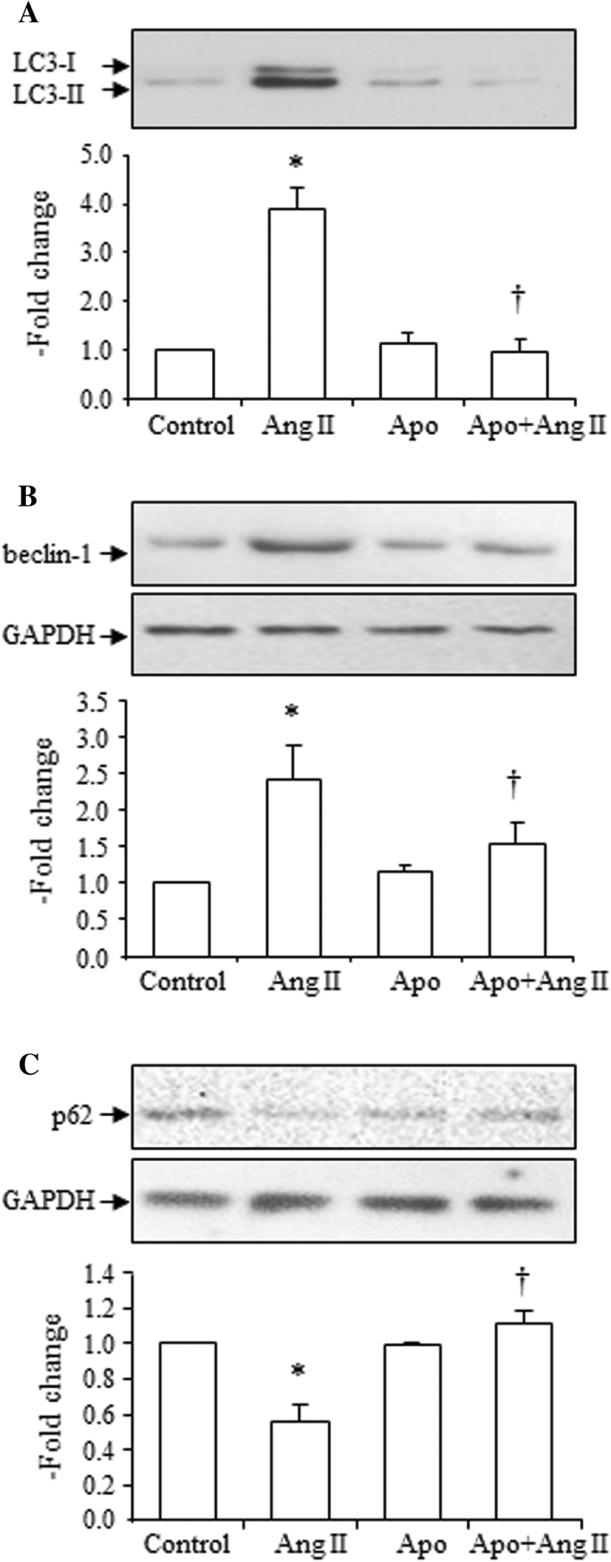
Effects of apocynin [Apo, 10^−4^ mol/L)] on Ang II (10^−7^ mol/L)-induced increase in LC3-II, increase in beclin-1 and decrease in SQSMT1/p62 expressions. **a** Effects of Apo on Ang II-induced increase of the LC3-II to LC3-I ratio, *top* representative blots of LC3-II and LC3-I after treatment with Ang II, *bottom* densitometry analysis of the ratio of LC3-II to LC3-I expression. **b** Effects of Apo on Ang II-induced increase of beclin-1 expression, *top* representative blots of beclin-1 and GAPDH expression after treatment with Ang II, *bottom* densitometry analysis of beclin-1 and GAPDH expression. **c** Effects of Apo on Ang II-induced decrease in SQSMT1/p62 expression, *top* representative blots of SQSMT1/p62 and GAPDH expression after treatment with Ang II, *bottom* densitometry analysis of SQSMT1/p62 and GAPDH expression. Control group is expressed as 1. Data are presented as mean ± SEM (*n* = 5–6). *p < 0.05 vs. control. ^†^ *p* < 0.05 vs. Ang II

NADPH oxidase is one of the downstream signaling pathways of the AT1 receptor, and apocynin (inhibitor of NADPH oxidase) markedly inhibited the Ang II (10^−7^ mol/L)-induced increase in the LC3-II to LC3-I ratio (Fig. 6a), increase in beclin-1 expression (Fig. 5b), and decrease in SQSMT1/p62 expression (Fig. 6c). Apocynin alone had no effects on the LC3-II to LC3-I ratio and expression of beclin-1 and SQSMT1/p62 (Fig. 6a–c). These data suggest that Ang II-induced autophagy occurs via NADPH oxidase activation.

### Effects of 5-HD on Ang II-induced increase in the LC3-II to LC3-I ratio, beclin-1 expression, and decrease in SQSMT1/p62 expression, and effects of diazoxide on the LC3-II to LC3-I ratio, beclin-1 and SQSMT1/p62 expressions

**Fig. 7:**
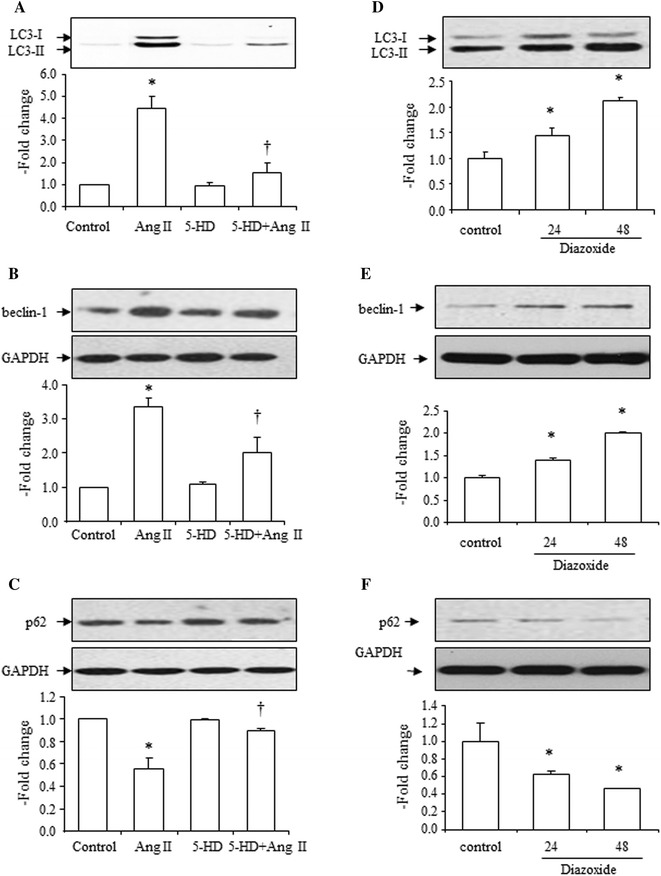
Effects of 5-HD (10^−4^ mol/L) on Ang II (10^−7^ mol/L)-induced increase in LC3-II, increase in beclin-1 and decrease in SQSMT1/p62 expression and effects of diazoxide (2 × 10^−4^ mol/L) on expression of LC3-II, beclin-1 and SQSMT1/p62. **a** Effects of 5-HD on Ang II-induced increase in the LC3-II to LC3-I ratio, *top* representative blots of LC3-II and LC3-I after treatment with Ang II, *bottom* densitometry analysis of the LC3-II to LC3-I ratio. **b** Effects of 5-HD on Ang II-induced increase in beclin-1 expression, *top* representative blots of beclin-1 and GAPDH expression after treatment with Ang II, *bottom* densitometry analysis of beclin-1 and GAPDH expression. **c** Effects of 5-HD on Ang II-induced decrease in SQSMT1/p62 expression, *top* representative blots of SQSMT1/p62 and GAPDH expression after treatment with Ang II, *bottom* densitometry analysis of SQSMT1/p62 and GAPDH expression. **d** Effects of diazoxide (24 and 48 h) on the LC3-II to LC3-I ratio, *top* representative blots of LC3-II and LC3-I expression after treatment with Ang II, *bottom* densitometry analysis of the LC3-II to LC3-I ratio. **e** Effects of diazoxide (24 and 48 h) on beclin-1 expression, *top* representative blots of beclin-1 and GAPDH expression after treatment with Ang II, *bottom* densitometry analysis of beclin-1 and GAPDH expression. **f** Effects of diazoxide (24 and 48 h) on SQSMT1/p62 expression, *top* representative blots of SQSMT1/p62 and GAPDH expression after treatment with Ang II, *bottom* densitometry analysis of SQSMT1/p62 and GAPDH expression. Control group is expressed as 1. Data are presented as mean ± SEM (*n* = 4–6). *p < 0.05 vs. control. ^†^ *p* < 0.05 vs. Ang II

The mitochondrial K_ATP_ channel inhibitor, 5-HD, suppressed Ang II-induced increases in the LC3-II to LC3-I ratio (Fig. 7a), increase in beclin-1 expression (Fig. 7b), and decrease in SQSMT1/p62 expression (Fig. 7c). 5-HD alone had no effect on the LC3-II to LC3-I ratio and expression of beclin-1 and SQSMT1/p62 (Fig. 7a–c).

Incubation with the K_ATP_ channel opener, diazoxide (for 24 and 48 h), also increased the LC3-II to LC3-I ratio (Fig. 7d), increased beclin-1 expression (Fig. 7e), and decreased SQSMT1/p62 expression (Fig. 7f).

These results revealed that Ang II-induced autophagy is mediated by mitochondrial K_ATP_ channels.

### Effects of SiRNA Atg5 on Ang II-induced increase in the LC3-II to LC3-I ratio, and decrease in SQSMT1/p62 expression

**Fig. 8:**
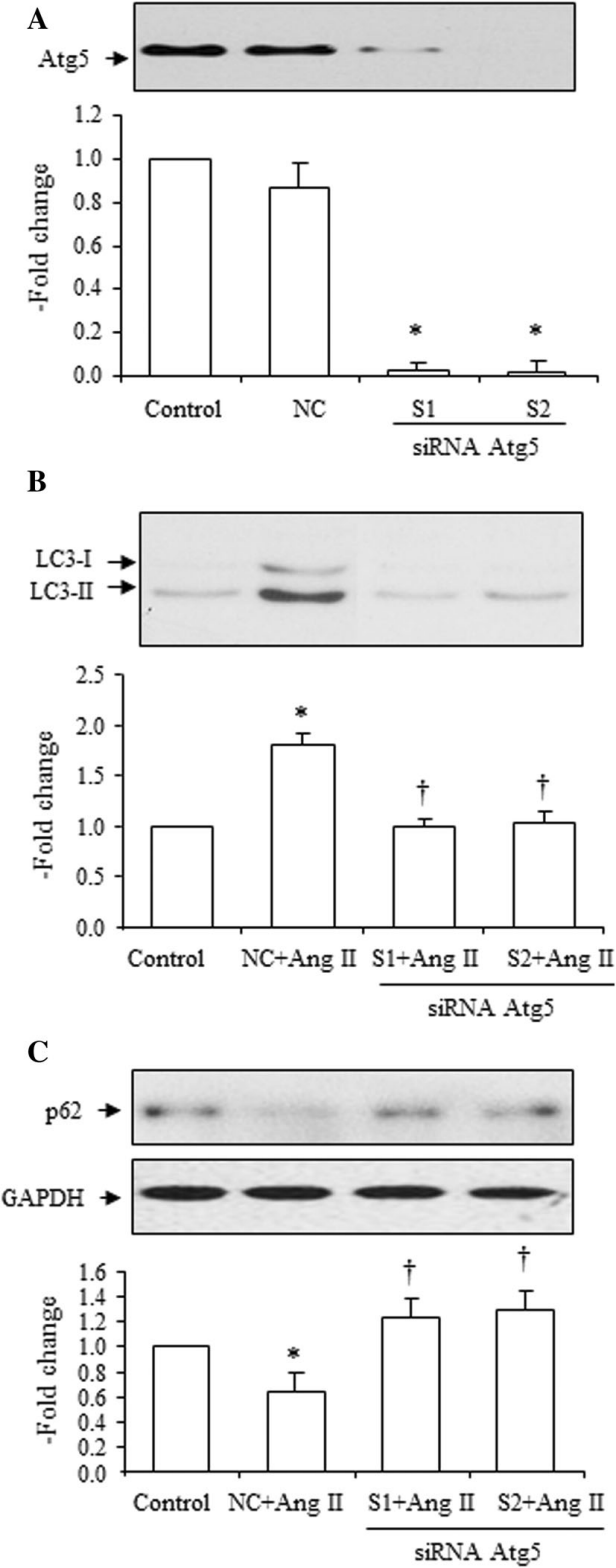
Effects of siRNA Atg5 on Ang II (10^−7^ mol/L)-induced increase in LC3-II, increase in beclin-1 and decrease in SQSMT1/p62 expression. **a** Effects of siRNA Atg5 on protein expression of Atg5. Two types of siRNA (S1 and S2) were utilized in the experiment, *top* representative blots of Atg5 expression after transfection, *bottom* densitometry analysis of Atg5 expression. **b** Effects of siRNA Atg5 (S1 and S2) on Ang II-induced increase in the LC3-II to LC3-I ratio, *top* representative blots of LC3-II and LC3-I expression after treatment with Ang II, *bottom* densitometry analysis of the LC3-II to LC3-I ratio. **c** Effects of siRNA Atg5 (S1 and S2) on Ang II-induced decrease of SQSMT1/p62 expression, *top* representative blots of SQSMT1/p62 and GAPDH expression after treatment with Ang II, *bottom* densitometry analysis of SQSMT1/p62 and GAPDH expression. *NC* indicates the negative control. The control group is expressed as 1. Data are presented as mean ± SEM (*n* = 6–8). *p < 0.05 vs. control. ^†^ *p* < 0.05 vs. Ang II

After Atg siRNA transfection, the expression of Atg5 in the treated cells was determined. Both types of oligos markedly decreased Atg5 expression (Fig. 8a), but the negative control oligo had no significant effect on Atg5 expression.

Both types of oligos reduced Ang II-induced increases in the LC3-II to LC3-I ratio (Fig. 8b), and decreases in SQSMT1/p62 expression (Fig. 8c).

### Ang II potentiated rapamycin-induced increase in the LC3-II to LC3-I ratio, beclin-1 expression, and decrease in SQSMT1/p62 expression

**Fig. 9:**
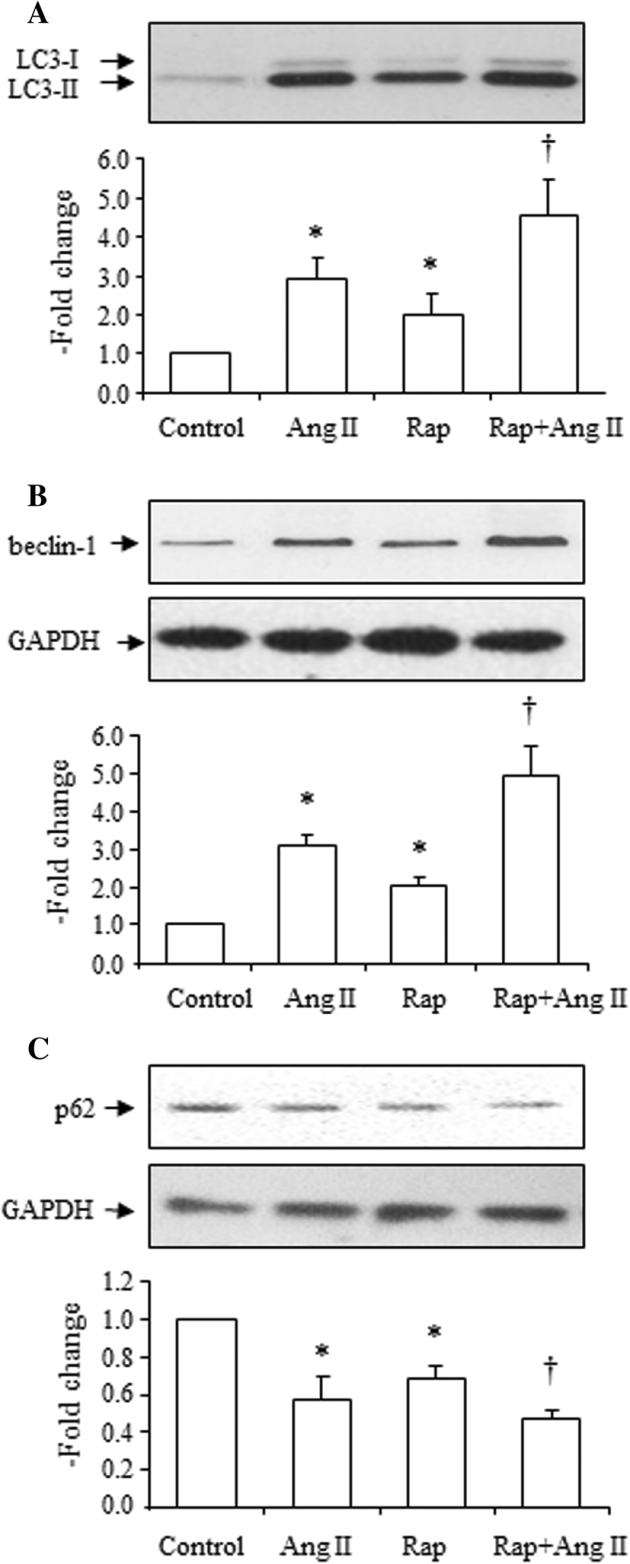
Effects of Ang II (10^−7^ mol/L) on rapamycin (Rap, 10 mg/L)-induced increase in LC3-II, increase in beclin-1 and decrease in SQSMT1/p62 expression. **a** Effects of Ang II on Rap-induced increase in the LC3-II to LC3-I ratio, *top* representative blots of LC3-II and LC3-I expression after treatment with Ang II, *bottom* densitometry analysis of the LC3-II to LC3-I ratio. **b** Effects of Ang II on Rap-induced increase of beclin-1 expression, *top* representative blots of beclin-1 and GAPDH expression after treatment with Ang II, *bottom* densitometry analysis of beclin-1 and GAPDH expression. **c** Effects of Ang II on Rap-induced decrease in SQSMT1/p62 expression, *top* representative blots of SQSMT1/p62 and GAPDH expression after treatment with Ang II, *bottom* densitometry analysis of SQSMT1/p62 and GAPDH expression. The control group is expressed as 1. Data are presented as mean ± SEM (*n* = 6–8). *p < 0.05 vs. control. ^†^ *p* < 0.05 vs. Ang II, and Rap

A well-known autophagy inducer, rapamycin, markedly increased the LC3-II to LC3-I ratio (Fig. 9a), increased expression of beclin-1 (Fig. 9b), and decreased expression of SQSMT1/p62 (Fig. 9c). Ang II potentiated the rapamycin-induced increase in the LC3-II to LC3-I ratio (Fig. 9a), increased beclin-1 (Fig. 9b), and decreased SQSMT1/p62 expression (Fig. 9c), confirming the effects of Ang II on autophagy in VSMCs.

### Effects of Ang II on formation of autophagosomes and effects of olmesartan, candesartan, apocynin, 5-HD, SiRNA Atg5 and rapamycin on Ang II-induced increase of autophagosomes

**Fig. 10:**
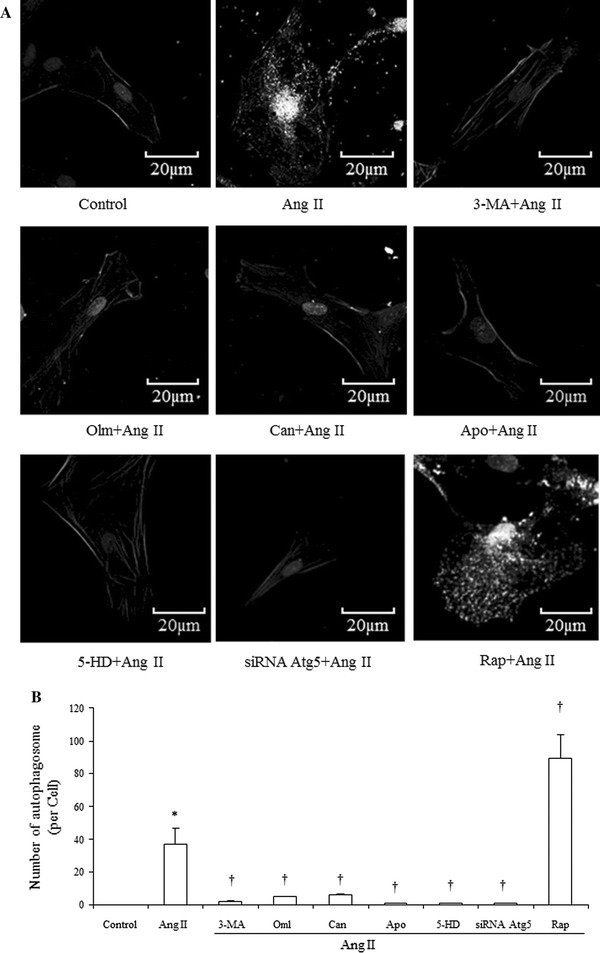
Immunofluorescent analysis of Ang II (10^−7^ mol/L) on the formation of autophagosomes and effects of Olm (10^−4^ mol/L), Can (10^−4^ mol/L), 3-MA (2 × 10^−4^ mol/L), Apo (10^−4^ mol/L), 5-HD (10^−4^ mol/L), siRNA Atg5 and Rap (10 mg/L) on Ang II-induced increase of autophagosomes. **a** Representative images of immunofluorescent detection of LC3-II. Nuclear, α-smooth muscle actin and LC3-II are stained with DAPI and corresponding antibodies as described in the method section. Images are shown in *gray scale*. *Bright dots* in the cell cytosol indicate autophagosomes. Magnification of the image is 400×. **b** Numbers of autophagosomes in VSMCs within each treatment group were counted. Data are presented as mean ± SEM (*n* = 8). *p < 0.05 vs. control. ^†^ *p* < 0.05 vs. Ang II

The autophagosome, which expresses the LC3-II protein, is one of the best markers for the presence of autophagy. Our results showed that Ang II stimulation for 24 h increased autophagosome numbers, which indicated an increase in autophagy in VSMCs (Fig. 10a, b). 3-MA (2 × 10^−3^ mol/L), Olmesartan (10^−4^ mol/L) and Candesartan (10^−4^ mol/L), apocynin (10^−4^ mol/L), 5-HD (10^−4^ mol/L) and Atg5 SiRNA all reduced Ang II-induced increases in autophagosomes. Meanwhile, Ang II potentiated rapamycin (10 mg/L)-induced increases in autophagosomes (Fig. 10a, b).

## Discussion

In this study, we have demonstrated that Ang II induces autophagy in a dose-dependent and time-dependent manner, and that this is mediated by the AT1 receptor and NADPH oxidase. Furthermore, our data indicates that mitochondrial K_ATP_ channels also play an important role in Ang II-induced autophagy in VSMCs. Our results provide novel understanding of Ang II association with cardiovascular diseases.

It is widely accepted that Ang II plays an important role in the development of cardiovascular diseases, and basic research and clinical application have confirmed the beneficial effects of ARB in the treatment of cardiovascular diseases [10, 17]. Numerous studies have revealed the mechanisms of Ang II-induced cell injury [7]. One of these mechanisms is through binding with AT1 receptor and activation of NADPH oxidase to produce ROS and thus trigger downstream signals resulting in cell apoptosis [13, 31, 45]. Our previous studies showed data that confirmed the above signal pathway [13, 31]. In addition, we were the first to demonstrate that mitochondrial K_ATP_ channels were involved in Ang II-induced downstream cell signaling, one of these signals plays a protective role in the pharmacological preconditioning effects of Ang II [19], while the other pathway has been demonstrated to play a cytotoxic role in Ang II-induced cell injury in VSMCs [18]. One important aspect of the role of mitochondrial K_ATP_ channels is that their opening leads to the depolarization of the mitochondria, resulting in a massive production of ROS to transduce the pro-apoptotic signal [18]. The deleterious role of ROS in diseases is strengthened by the observation that clinical application of antioxidant in various diseases has favorable outcomes [1, 11, 41]. In the present study, we have shown an increase of ROS in response to Ang II stimulation. Our data revealed that Ang II-induced autophagy is mediated by NADPH oxidase, which has been demonstrated to be involved in ROS generation to mediated downstream signal. It should be noted that several possible pathways have been reported to be involved in the activation of NADPH oxidase, one involves translocation of NADPH oxidase subunits (such as p47phox, p67phox and p40phox) to the membrane [47]. Another mechanism is mediated by increased protein expression of NADPH oxidase subunits or homologous proteins (such as gp91phox, Nox1) [24, 30, 47, 51]. Recently, Niu et al. [32] reported that the Nox activator 1, a functional homolog of p67phox, regulates redox signaling in VSMC. Although our present study has not shown which pathway is essential for Ang II-induced increase of ROS generation, it may involve the increased expression of several subunits like Nox1, Nox4 or Nox activator 1. Further studies are needed to clarify this issue.

Autophagy, or cellular self-digestion, primarily serves as a protective mechanism to prolong cell survival under conditions of stress [22, 39]. Autophagy participates in the turnover of damaged mitochondria and other cellular organelles [49]. Furthermore, autophagy is involved in the clearance of polyubiquitinated protein aggregates, which are accumulated during stress, aging and diseases due to perturbation of protein structure or folding [23]. Autophagy has also been implicated in lipid metabolism [44], protection from apoptotic injury [4, 38] and protection from neurodegeneration [28]. Other studies, however, have demonstrated that autophagy can result in cell death, termed programmed cell death type II in cerebellar granule cells and other cell lines [6, 9]. In the cardiovascular system, modulations in autophagy have been associated with heart diseases, including cardiomyopathies, cardiac hypertrophy, ischemic heart disease, heart failure, and ischemia–reperfusion injury [20]. In the vasculature, it has been reported that increased numbers of autophagosomes are evident in macrophages from atherosclerotic plaques [48]. Autophagy may stabilize atherosclerotic plaques by preventing macrophage apoptosis and plaque necrosis and by preserving efferocytosis [26]. Although the existing evidence shows inconsistent conclusions in the role of autophagy in human diseases, much of these data are dependent upon the conditions, of cells themselves. Autophagy plays a protective role under physiological conditions, but it may contribute to t pathogenesis in response to stress by creating a condition of unrestrained autophagic activity.

Molecular regulation of autophagy and its role in human diseases have recently been reviewed [8]. The complicated process of autophagy requires two ubiquitin-like conjugation systems: the ATG5-ATG12 conjugation system and the LC3-ATG8 conjugation system to elongate the autophagosome. The conversion of a cytosolic truncated form of LC3 (LC3-I) to its autophagosomal membrane-associated, phosphatidylethanolamine-conjugated form (LC3-II), visible as discrete points on immunofluorescent analysis, indicates autophagosome formation as stated by Choi et al. [8]. In addition, according to the recent guidelines for monitoring autophagy, it is also essential to detect the autophagic flux to accurately ascertain the induction of autophagy [19]. In the present study, we observed an increase in LC3-II and beclin-1 expression, and a decrease in SQSMT1/p62 expression, both of which are the markers of autophagic flux [21, 29]. This observation of autophagic flux confirmed Ang II-induced autophagy, which is consistent with previous observations in podocytes [54].

An increasing number of investigations are now focused on the role of Ang II-induced autophagy in the heart. It has been reported that Ang II increases autophagosome formation via the Ang II type I (AT1) receptor and that this response is constitutively antagonized by co-expression of the AngII type 2 (AT2) receptor in neonatal cardiomyocytes [36]. Several reports have demonstrated that autophagy is involved in Ang II-induced cardiac injury, such as remodeling [34], inflammation [33], and post-burn dysfunction [53]. Although the pathogenic effects of Ang II on the vascular system such as promoting atherosclerosis are well documented, there is still no report focusing on the role of Ang II-induced autophagy in the vascular system. Whether Ang II-induced autophagy potentiates the development of atherosclerosis, or it can stabilizes formed plaques is still unknown. Although our present data have not shown the evidence that Ang II-induced autophagy is involved in the development of atherosclerosis or stabilization of plaque, we speculate that Ang II-induced autophagy may play a role in the onset of vascular injury. This may include not only the apoptosis of endothelial cells, but may also include cell autophagy (VSMC) via the induction of autophagy-related cell calcification or migration. Future work is still needed to explore the detailed mechanism of Ang II-induced autophagy and its role in vascular injury.

**Fig. 11:**
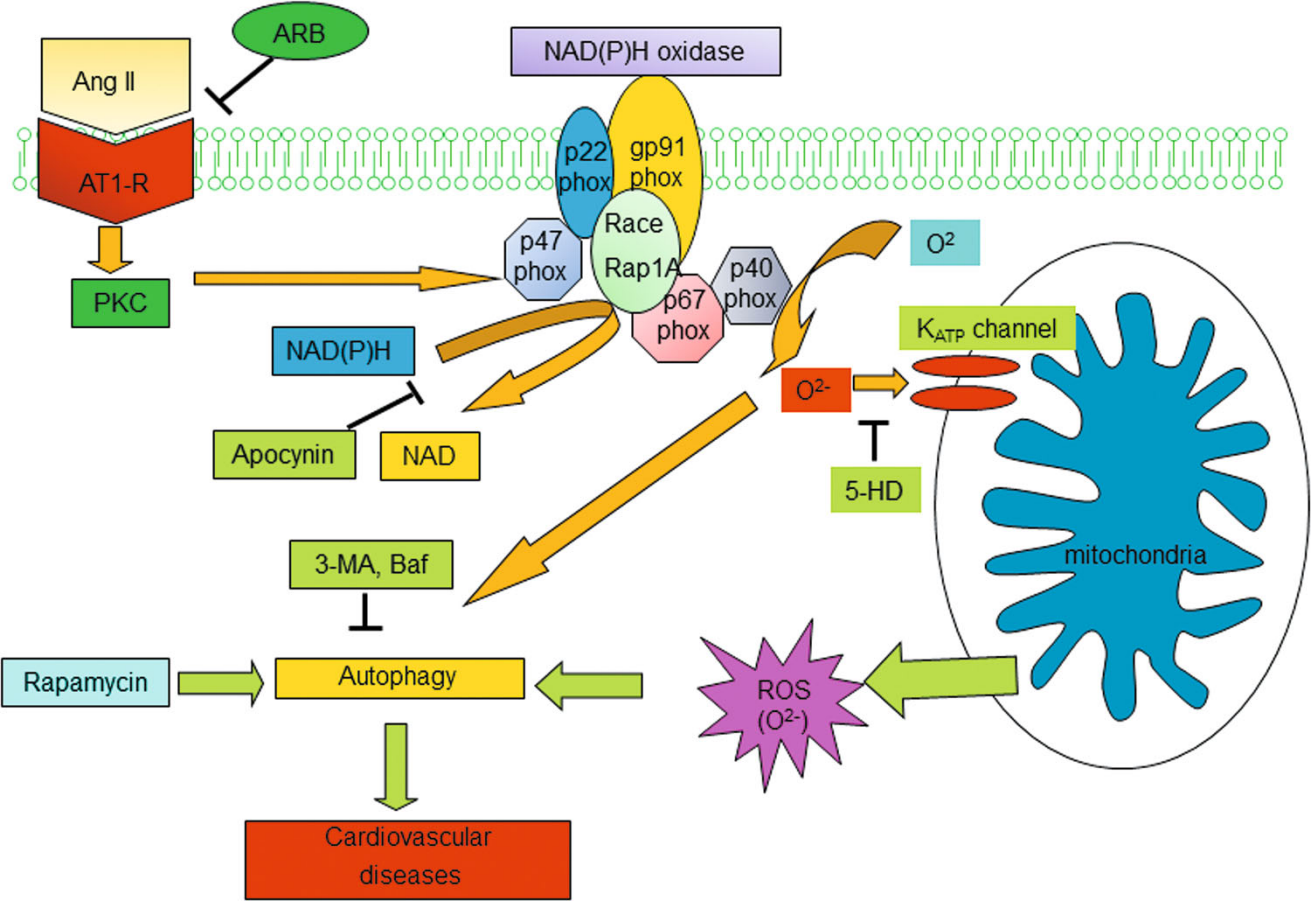
Possible mechanisms of Ang II-induced autophagy. Ang II binds to Ang II type I receptors (AT1-R), which activates NADPH oxidase to induce superoxide (O_2_^−^) generation. Mitochondrial K_ATP_ channels can be activated by O_2_^−^ to produce more generation of reactive oxygen species (ROS) to induce autophagy. Angiotensin II receptor blocker (ARB), NAD(P)H oxidase inhibitor (Apocynin), mitochondrial K_ATP_ channel inhibitor (5-HD)

In conclusion, our present study revealed a novel pathway of Ang II effects on VSMCs which results in Ang II-induced cell autophagy through the AT1 receptor, NADPH oxidase and mitochondrial K_ATP_ channels (Fig. 11). Our study may provide a better understanding on the effects of Ang II in the cardiovascular system, yielding additional therapeutic targets for human diseases.
